# Empyema Treated by Injections

**Published:** 1858-02

**Authors:** David Prince

**Affiliations:** Jacksonville, Ill.


### EMPYEMA TREATED BY INJECTIONS.

BY DAVID PRINCE, M. D., OF JACKSONVILLE, ILL.

The perusal of Dr. Brainard's case of cure of empyema of the pleural cavity by injections of iodine (*N.-W. M and S. J.* for November), has induced me to record a case of my own in your valuable Journal.

Little Henry Lurton, aged four years, the third child of healthy parents, and of healthy constitution himself, had an attack of pleuritis of the right side, and was left entirely to the aids of the *vis medicatrix naturæ,* under the guise of infinitesimal doses for about six weeks, when, on the 28th of January, 1856, I was called upon for surgical interference.

At this time, the enlargement of the right side was very obvious to the eye, and the sound was dull on percussion.

Frequent and quick pulse and daily fever and nightly sweats were present, while the little patient could breathe only in the sitting posture, and his sleep was in the rocking chair.

A considerable quantity of pus was discharged through the canula left in the orifice made by the trochar, giving immediate and inexpressible relief.

*Feb. 9th.*—The puncture had not been kept open, and the demand for a second puncture became urgent. The amount discharged was somewhat smaller than before. The opening was from this time prevented from closing by the daily introduction of a tent, upon the withdrawing of which, a considerable quantity of ill-conditioned pus would be discharged.

As nature seemed not to be making good progress with the case, it was resolved to see if the behavior of iodine would be as good here as in other cavities. On the 25th of March, two drachms (ʒij) of strong tincture of iodine were thrown in by a small glass syringe. This was done after the tent had been withdrawn, and the pus collected during the preceding twenty-four hours had pretty well escaped; the tent was again introduced as usual, to be daily withdrawn as before.

The injection was repeated at intervals of about a week, for five times, when the discharge had pretty much ceased. The respiratory murmur could be heard throughout the whole extent of the lung; all sign of disease had disappeared, and by the middle of May the boy was at play with his top.

